# A neuronal DNA damage response is detected at the earliest stages of Alzheimer's neuropathology and correlates with cognitive impairment in the Medical Research Council's Cognitive Function and Ageing Study ageing brain cohort

**DOI:** 10.1111/nan.12202

**Published:** 2015-04-23

**Authors:** Julie E. Simpson, Paul G. Ince, Fiona E. Matthews, Pamela J. Shaw, Paul R. Heath, Carol Brayne, Claire Garwood, Adrian Higginbottom, Stephen B. Wharton, MRC Cognitive Function and Ageing Neuropathology Study Group

**Affiliations:** ^1^ Sheffield Institute for Translational NeuroscienceUniversity of SheffieldSheffieldUK; ^2^ MRC Biostatistics UnitUniversity of CambridgeCambridgeUK; ^3^ Institute of Public HealthUniversity of CambridgeCambridgeUK

**Keywords:** cognitive impairment, DNA damage response, DNA‐PKcs, neurone, γH2AX

## Abstract

**Aims:**

Population‐based studies have shown that approximately 20% of the ageing population (aged 65 years and over) with dementia have little or no classical Alzheimer‐type neuropathology. Cumulative DNA damage and a reduced capacity of DNA repair may result in neuronal dysfunction and contribute to cognitive impairment independent of Alzheimer‐type pathology in the ageing brain.

**Methods:**

We investigated expression of the DNA damage response (DDR)‐associated molecules γH2AX and DNA‐PKcs using immunohistochemistry and western blotting, and senescence‐associated β‐galactosidase in the frontal association neocortex of cases with low levels of Alzheimer‐type pathology (Braak & Braak stage 0–II), and explored their relationship to cognitive impairment in a population‐representative sample from the Medical Research Council's Cognitive Function and Ageing Study cohort.

**Results:**

Increases in both γH2AX ^+^ (*r*_s_ = −0.36, *P* = 0.025) and DNA‐PKcs ^+^ (*r*_s_ = −0.39, *P* = 0.01) neuronal counts were associated with a lower Mini‐Mental State Examination score. Increasing levels of senescence associated‐β‐gal^+^ pyramidal neurones were weakly associated with the total number of DNA‐PKcs^+^ neurones (*P* = 0.08), but not with traditional senescence‐associated signalling molecules, including p53 and p16.

**Conclusion:**

The association between the neuronal DDR and cognitive impairment, independent of AD pathology in the ageing brain, may be suggestive of a causal link via neuronal dysfunction.

## Introduction

Alzheimer's disease (AD), the most common clinically diagnosed subtype of dementia in the ageing population, is classically characterized by extracellular deposits of β‐amyloid and intracellular neurofibrillary tangles of hyperphosphorylated tau. Population‐based studies have shown that approximately 20% of individuals with dementia have little or no tangle pathology [1]. As life expectancy continues to increase, so the burden of cognitive impairment increases and with it a growing need to identify, prevent and effectively treat dementia. Despite large numbers of studies investigating the complex molecular mechanisms underlying dementia, recent clinical trials have failed to show efficacy [2]. There is a compelling case to identify other potential factors contributing to neuronal dysfunction and cognitive decline, either independent of or interacting with classical AD lesions.

The Medical Research Council's Cognitive Function and Ageing Study (CFAS) is a large, well characterized, population‐representative, prospective, longitudinal study of the ageing population in the United Kingdom that facilitates unbiased assessment of relationships between pathology and dementia [1, 3, 4]. As cases are not pre‐selected according to clinical groups, population‐based studies are free from sampling bias and are essential for understanding the association between disease and pathology, providing complementary information to more classical case–control designs [5]. The population approach of CFAS has thus generated a number of insights into the biological basis of dementia in a population setting, as reviewed [4, 6]. CFAS has demonstrated an extensive overlap of Alzheimer‐type pathology (neurofibrillary tangles and plaques) among demented and non‐demented individuals [1], which increases at the oldest ages [7]. This finding is supported by results from non‐population‐representative studies [8, 9] and suggests that factors other than classical pathologies contribute to neuronal dysfunction and cognitive impairment. However, the mechanisms that underlie neuronal vulnerability, and which may initiate adverse cellular processes many years before eventual cognitive decline, are not fully understood and likely highly complex. Recently, it has been proposed that processes that characterize normal ageing, including mitochondrial dysfunction, telomere attrition and cellular senescence, are interconnected and may contribute to neurodegeneration [10]. These processes need to be explored in detail in the ageing brain in order to fully understand relevant mechanisms of neuronal dysfunction and cognitive impairment.

Oxidative stress is a feature of brain ageing that results in the oxidative modification of proteins, lipids, DNA and RNA [11, 12] and plays a key role in several neurodegenerative pathologies, including AD, Parkinson's disease and motor neurone disease [13, 14, 15]. Oxidative damage to nucleic acids is evident in mild cognitive impairment (MCI) and early AD [16, 17, 18], suggesting that oxidative stress is an early contributor to neuronal dysfunction which, either independently of or interacting with early Alzheimer‐type pathology, results in cognitive impairment.

Oxidative DNA damage, in particular DNA double‐strand breaks, is a potent inducer of a DNA damage response (DDR), which is characterized by the activation of sensor kinases, including DNA‐protein kinase catalytic subunit (DNA‐PKcs), and regions of DNA damage incorporating phosphorylated histone γH2AX [19]. Chronic oxidative stress, a persistent DDR and telomere dysfunction have all been implicated in the irreversible arrest of the cell cycle resulting in cellular senescence [20]. Although senescence is usually defined in the context of proliferation arrest, a broader senescent phenotype is being defined with cell‐cycle arrest as one component that may not be essential to the definition. Recent studies of brain ageing have demonstrated senescent human astrocytes [21, 22] and senescent mouse neurones [23] and suggest that cells with a senescent phenotype may contribute to dysfunction of the ageing brain. Activation of senescence‐associated β‐galactosidase, a widely accepted general marker of the senescent phenotype, is downstream of p53 and p21 in ageing mice [23]. To date, the presence of senescent neurones and the signalling pathway(s) involved in post‐mitotic neuronal senescence in the human brain are unknown.

Previous investigation of the CFAS cohort has demonstrated that high levels of oxidative stress and DNA damage occur at the lowest Braak and Braak neurofibrillary tangle stages, indicating that oxidative stress and the DDR are not a late stage effect of established Alzheimer‐type pathology and may contribute to the development and progression of age‐related neurodegeneration [22]. We hypothesized that, within an ageing population of individuals with little AD neuropathology, high levels of oxidative stress and neuronal DDR might contribute to neuronal dysfunction and cognitive impairment. Therefore, in this study, we defined the population variation in oxidative stress (lipid peroxidation) and the neuronal DDR (γH2AX and DNA‐PKcs) in the frontal association neocortex of CFAS cases with low levels of Alzheimer‐type pathology, pre‐selected only by being at low (0–II) Braak and Braak neurofibrillary tangle stage and investigated the relationship to dementia status.

## Materials and methods

### Human central nervous system (CNS) tissue

CNS tissue was obtained from MRC CFAS, following Research Ethics Committee approval (REC ref 12/EM/0118). Brains were donated as part of the CFAS study from consented individuals. The structure of the CFAS neuropathology cohort has been previously described and reviewed [3, 4]. Brains were dissected following a standard protocol [24]. One cerebral hemisphere was fixed in formalin for a minimum of 4 weeks, and the other hemisphere sliced in the coronal plane, rapidly frozen and stored at −80°C. Neuropathological lesions were assessed as part of the core CFAS neuropathology study using a modified protocol from the Consortium to Establish a Registry of Alzheimer's Disease [25] (http://www.cfas.ac.uk). Braak and Braak staging for neurofibrillary tangle stage was assessed by analysis of AT8 (detects tau phosphorylated at serine 199/202) immunostained sections of hippocampus and isocortical regions [26]. This scheme identifies six stages of neurofibrillary tangle progression, with hierarchical involvement of entorhinal (stages I–II), limbic (stages III–IV) and neocortical (stages V–VI) regions. The core neuropathology assessment in CFAS also included semi‐quantitative assessment of diffuse and neuritic plaques, vascular pathology, Lewy body pathology and other identified lesions.

This study investigated Braak and Braak stage 0–II cases from the CFAS cohort. Of this cohort, frozen frontal cortex (FCx) was available for 39 cases from two CFAS centres, one case with Pick's disease was excluded on review of the neuropathology, meaning 38 cases with an average age at death of 82 years (range 70–102 years), median post mortem delay of 19.2 h (IQR 18–24 h) and tissue pH 6.6 (IQR pH 6.3–6.9) were used in the study. A summary of the main neuropathological features of the cohort is shown in Table 1.

**Table 1:** Neuropathology of frontal cortex from low Braak and Braak cases

Pathology	No dementia	Dementia
Braak stage		
0	2	0
I	6	2
II	14	11
Neuritic plaques		
None	18	10
Mild	2	2
Moderate	2	1
Diffuse plaques		
None	11	5
Mild	5	4
Moderate	6	1
Severe	0	3
Parenchymal CAA		
None	20	11
Mild	1	2
Meningeal CAA		
None	19	10
Mild	2	0
Moderate	0	2
Infarcts*		
Absent	18	7
Present	4	5
Haemorrhage*		
None	21	12
Small vessel disease*		
Absent	11	2
Present	11	11

*Based on global pathology scores from all assessed brain regions. No cases in either group displayed neuronal loss, ballooned neurones, severe gliosis, Lewy bodies, Pick bodies or spongiform changes.

CAA, cerebral amyloid angiopathy.

### Dementia status

Individuals in the study were regularly interviewed and underwent Geriatric Mental State‐Automated Geriatric Examination for Computer‐Assisted Taxonomy and Mini‐Mental State Examination (MMSE) [1, 7], the interval between last MMSE and death was 1.6 years, median 1.2 years. Dementia status at death was determined on the basis of all information available for each participant [1, 27]. Within this cohort, 13 participants had dementia, 22 had no dementia and three participants had an unknown dementia status at death due to the lack of information in the years preceding death.

### Western blot analysis

FCx samples were homogenized in Tris extraction buffer (10 mM Tris‐HCl pH 7.4, 0.8 M sodium chloride, 1 mM ethylene glycol tetraacetic acid (EGTA), 10% sucrose, 0.1 mM phenylmethanesulphonylfluoride (PMSF), 2 μg/ml aprotonin, 10 μg/ml leupetin, 5 μg/ml pepstatin, 40 mM β‐glycerophosphate, 50 mM sodium fluoride, 200 μM sodium orthovanadate) and centrifuged at 14 000 rpm for 30 min at 4°C. The protein content of the supernatant was measured using the bicinchoninic acid method and equal protein amounts (20 μg) analysed by western blot analysis. Proteins were separated by sodium dodecyl sulphate‐polyacrylamide gel electrophoresis (15% for γH2AX, 8% for malondialdehyde (MDA) and 6% for DNA‐PKcs) and transferred to nitrocellulose (BioRad Laboratory, Hemel Hempstead, UK). Membranes were incubated overnight with rabbit anti‐γH2AX (1:1000; R&D Systems, UK), rabbit anti‐MDA primary antibody (1:1000; Cell Biolabs, Cambridge, UK) or mouse anti‐DNA‐PKcs (1:500; Calbiochem, UK), followed by the appropriate horseradish peroxidise‐linked secondary antibody (1:1000) and visualized by enhanced chemiluminescence detection. To confirm equal protein loading, the membrane was reprobed for β‐actin (1:5000; AbCam, Cambridge, UK). Protein expression levels were determined by densitometry of the appropriate, sub‐saturated band using G:BOX Chemi (Syngene, Cambridge, UK) and the results normalized to β‐actin.

### Immunohistochemistry

Immunostaining was performed using a standard avidin biotinylated enzyme complex method, and the signal visualized with diaminobenzidine (Vector Laboratories, Peterborough, UK). A summary of the primary antibodies used and their conditions for use is shown in Table 2.

**Table 2:** Antibody source and specificity

Antibody	Isotype	Dilution (time, temp)	Supplier
DNA‐PKcs	Mouse IgG_1_	1:400 (60 min, RT)	Calbiochem, UK
γH2AX	Rabbit IgG	1:2000 (60 min, RT)	R&D Systems, UK
caspase‐3	Rabbit IgG	1:100 (60 min, RT)	AbCam, UK
p16 (INK4)	Mouse IgG	Prediluted (o/n, 4°C)	BioGenex, UK
p53	Mouse IgG_2A_	1:100 (60 min, RT)	Santa Cruz, UK

o/n, overnight; RT, room temperature.

### Quantification of γH2AX ^+^ and DNA‐PKcs ^+^ pyramidal neurones

Image capture of three adjacent 350 μm‐wide cortical ribbons through the full thickness of the FCx was performed using Cell R software (Olympus Biosystems, Watford, UK), using a × 20 objective. The image was automatically thresholded and the total number of positive nuclei was quantitated using Analysis^˄^D software (Olympus Biosystems, Watford, UK). The number of DNA‐PKcs^+^ or γH2AX^+^ positive pyramidal neuronal nuclei was determined using a size exclusion of >250 pixels, and the number of positive small nuclei (glia and small neurones) determined by subtracting the number of pyramidal neuronal nuclei from the total number of positive nuclei. To assess the total number of pyramidal neurones and small cells in each case, an identical quantitation protocol was performed on haematoxylin‐only stained serial sections. Therefore, in addition to total counts of immunopositive neurones, the proportion of immunopositive neurones was also determined by dividing the number of positive neurones by the total number of neurones in the FCx.

### Semi‐quantitative assessment of p53^+^ and activated caspase‐3^+^ pyramidal neurones

The number of p53^+^ neuronal nuclei was rated semi‐quantitatively using the × 20 objective as follows: no positive neuronal nuclei (none), 1–5 positive neuronal nuclei (low), 5–20 positive nuclei (medium) and >20 positive nuclei (high). Activated caspase‐3^+^ pyramidal neurones were rated semi‐quantitatively as follows: no immunoreactive neurones (low), 1–5 positive neurones (medium), >5 positive neurones (high).

### Senescence‐associated β‐galactosidase (SA‐β‐gal) activity

SA‐β‐gal activity was assessed using a senescence cells histochemical staining kit (Sigma, Poole, UK). Briefly, 10 μm frozen sections were fixed with 2% paraformaldehyde for 6 min at RT. The sections were washed three times with phosphate buffered saline (PBS) for 10 min at RT and incubated overnight with freshly prepared SA‐β‐gal staining solution (pH 6) at 37°C in a humid chamber. The sections were washed three times with PBS for 10 min at RT, counterstained with nuclear fast red, dehydrated through a graded series of ethanol and cleared in xylene. Neuronal expression of SA‐β‐gal was rated semi‐quantitatively using the × 20 objective in the cortical region of most intense stain, as follows: no positive neurones (0); 1–5 positive cells (1); 5–20 positive cells (2); >20 positive neurones (3).

### Statistical analysis

Due to the small number of individuals, all tests were undertaken using non‐parametric methods but checked for consistency using parametric regression models. Spearman's correlation coefficient was used to test associations between continuous variables, with Mann–Whitney *U*‐Test, Kruskal–Wallis test for groups and non‐parametric trend test for ordered categories. SA‐β‐gal^+^ neurones were quantified by two independent observers (JES and SBW) and the inter‐rater reliability assessed by calculating Cohen's kappa coefficient. Analyses were performed using STATA version 13 (StataCorp LP, College Station, Texas, USA) and IBM® SPSS® Statistics 21 (Armonk, NY, USA).

## Results

### Population variation in oxidative stress and a neuronal DDR

**Figure 1:**
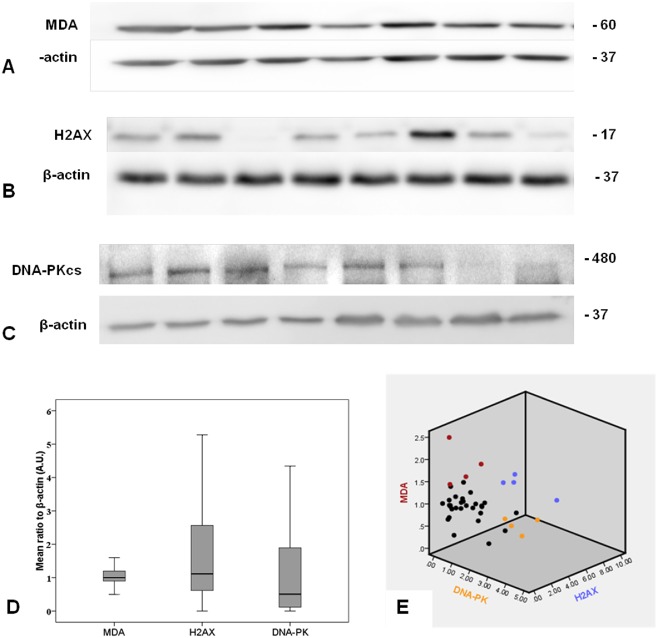
Population variation in lipid peroxidation and a DNA damage response. Representative western blot of (**A**) malondialdehyde, (**B**) γH2AX and (**C**) DNA‐PKcs expression in frontal cortex homogenates from Braak 0–II cases. (**D**) Densitometric analysis of all cases were normalized to β‐actin and expressed in densitometric arbitrary units (AUs). (**E**) 3D‐scatterplot of protein levels. Cases with the highest levels of MDA, γH2AX and DNA‐PKcs and are shown in red, blue and yellow, respectively.

The population variation in lipid peroxidation and a DDR was established using western blotting for MDA, γH2AX and DNA‐PKcs in total protein extracts from FCx (Figure 1 **A–D**, Table 3). Both MDA and γH2AX detected single bands at 60 kDa and 17 kDa, respectively. Multiple bands were detected in the DNA‐PKcs immunoblot that may represent DNA‐PKcs degradation products, therefore only the band at 480 kDa representing intact DNA‐PKcs was quantitated. MDA, DNA‐PKcs and γH2AX protein levels did not associate with each other and individuals with the highest values for each marker did not correspond (Figure 1 **E**), suggesting that these markers behave differently. Neither MDA nor γH2AX detection by western blotting showed a relationship to brain pH or post mortem delay (PMD) (all *P* > 0.2). Similarly, DNA‐PKcs was not associated with PMD but did show a significant association with pH (*r*_s_ = 0.50, *P* = 0.0012).

**Table 3:** Population variation in the expression of malondialdehyde and the DNA damage‐related molecules γH2AX and DNA‐PKcs at the earliest stages of Alzheimer's neuropathology, as detected by western blotting. Protein expression levels were determined by densitometry and normalized to β‐actin (densitometric arbitrary units)

	MDA	γH2AX	DNA‐PKcs
Number	38	38	38
Mean (SD)	1.08 (0.39)	1.99 (2.14)	1.21 (1.35)
Median (IQR)	1.0 (0.8–1.2)	1.15 (0.57–2.72)	0.54 (0.12–2.02)

IQR, inter‐quartile range; SD, standard deviation.

**Figure 2:**
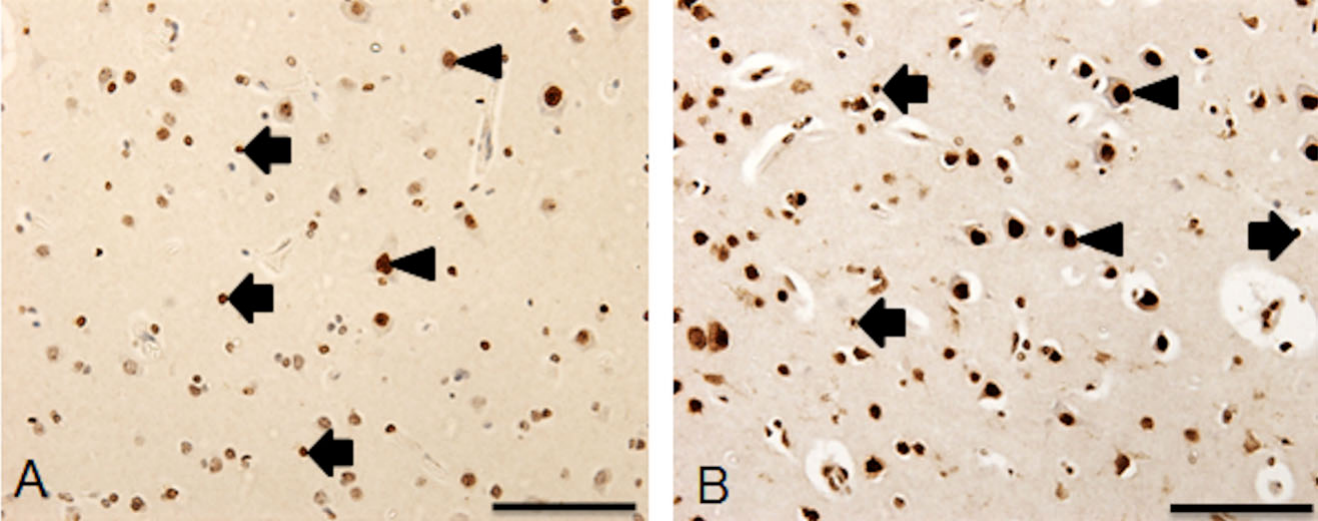
Expression of DNA damage‐related molecules. (**A**) γH2AX and (**B**) DNA‐PKcs expression was associated with the nuclei of small glia (arrow) and larger pyramidal neurones (arrow head). Scale bar represents 100 μm.

To determine whether oxidative stress and DNA damage may result in processes leading to neuronal dysfunction and injury, we sought to define the DDR in neurones using immunohistochemistry. Specific nuclear immunostaining of γH2AX and DNA‐PKcs was associated with cells morphologically resembling pyramidal neurones and glia (Figure 2 **A,B**). The number and proportion of γH2AX^+^ and DNA‐PKcs^+^ neurones is shown in Table 4. The number of γH2AX^+^ neurones significantly related to the number of DNA‐PKcs^+^ neurones (*r*_s_ = 0.35, *P* = 0.03), though this affect was attenuated for the proportion of positive neurones (*r*_s_ = 0.22, *P* = 0.19) as shown in Figure 2 **C**. No relationship was seen between γH2AX^+^ or DNA‐PKcs^+^ neurones and MDA western blot data, in either the number or proportion of the immunopositive neurones (all *P* values > 0.1).

**Table 4:** Number and proportion of γH2AX ^+^ and DNA‐PKcs ^+^ neurones in the FCx at the earliest stages of Alzheimer's neuropathology

	Neurones	γH2AX Count	Proportion	DNA‐PKcs Count	Proportion
Mean (SD)	97.6 (9.3)	37.6 (19.5)	0.38 (0.19)	51.6 (24.4)	0.52 (0.23)
Median (IQR)	97.6 (91.3–103.5)	33.5 (29.1–44.1)	0.38 (0.28–0.48)	49.8 (35.3–69.8)	0.52 (0.38–0.66)

IQR, inter‐quartile range; SD, standard deviation.

### Neuronal DDR is associated with a lower final MMSE score

**Figure 3:**
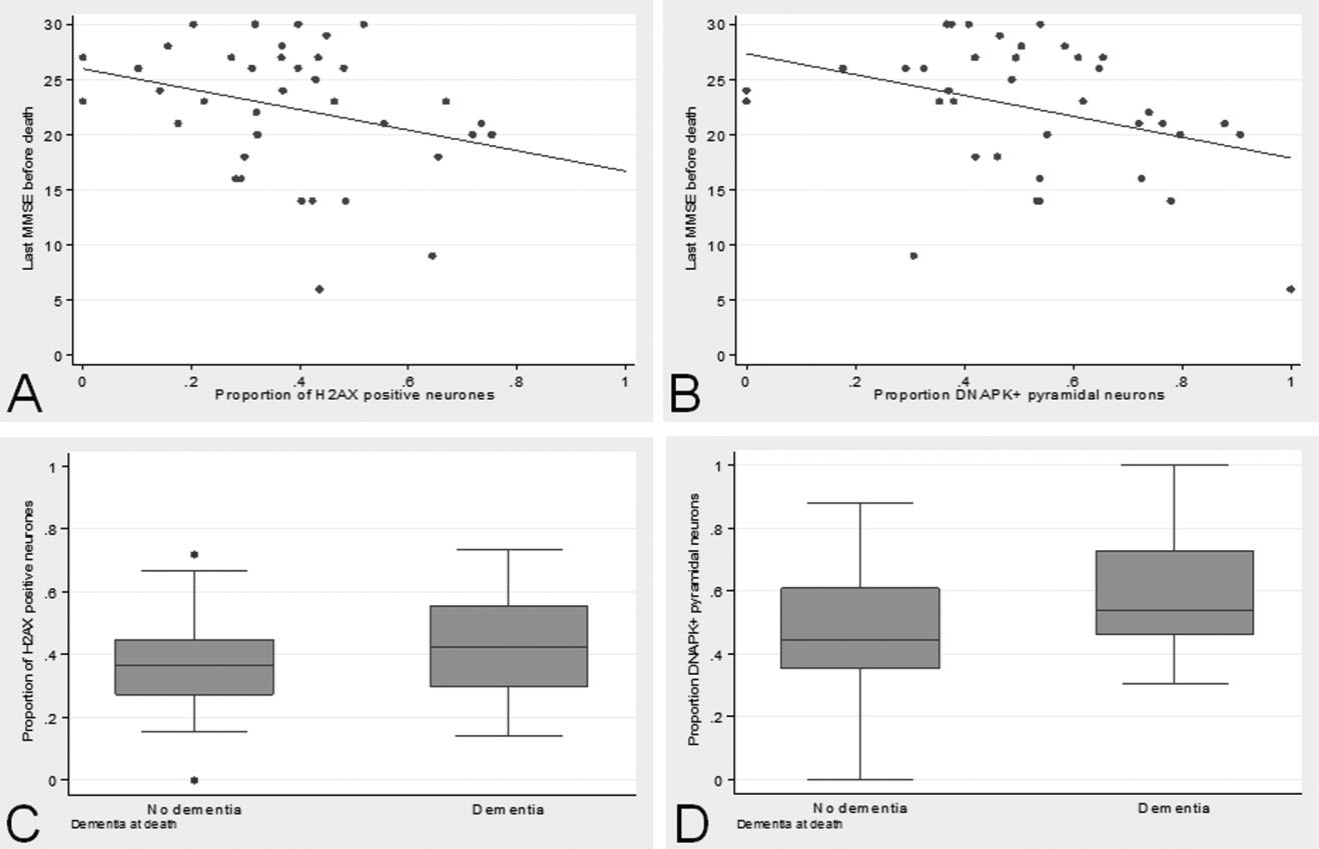
Association of the neuronal DDR with cognitive impairment. Both (**A**) γH2AX ^+^ and (**B**) DNA‐PKcs ^+^ neurones negatively associated with the patients’ last MMSE score. (**C**) γH2AX ^+^ and (**D**) DNA‐PKcs ^+^ neurones were higher in cases with dementia but did not reach significant levels.

Both γH2AX^+^ (*r*_s_ = −0.36, *P* = 0.025) and DNA‐PKcs^+^ (*r*_s_ = −0.39, *P* = 0.018) neuronal counts and proportions (*r*_s_ = −0.29, *P* = 0.07 and *r*_s_ = −0.35, *P* = 0.03) were significantly related to the patients’ last MMSE score (Figure 3 **A,B**); these results were unaffected by length of time between last measured MMSE and death. Levels of γH2AX^+^ and DNA‐PKcs^+^ neurones were higher in cases with dementia but did not reach significant levels (*P* = 0.11 for γH2AX^+^ neuronal counts, *P* = 0.31 for proportion of γH2AX^+^ neurones, *P* = 0.08 for DNA‐PKcs^+^ neuronal counts, *P* = 0.11 for proportion of DNA‐PKcs^+^ neurones) (Figure 3 **C,D**). Cases with high γH2AX^+^ or DNA‐PKcs^+^ neuronal counts (or proportion of immunopositive neurones) were more likely to have a lower final MMSE score than cases with lower γH2AX^+^ or DNA‐PKcs^+^ neurones.

### No association between neuronal DDR and age, sex, ApoE genotype, vascular pathology, diffuse or neuritic β‐amyloid plaques

**Figure 4:**
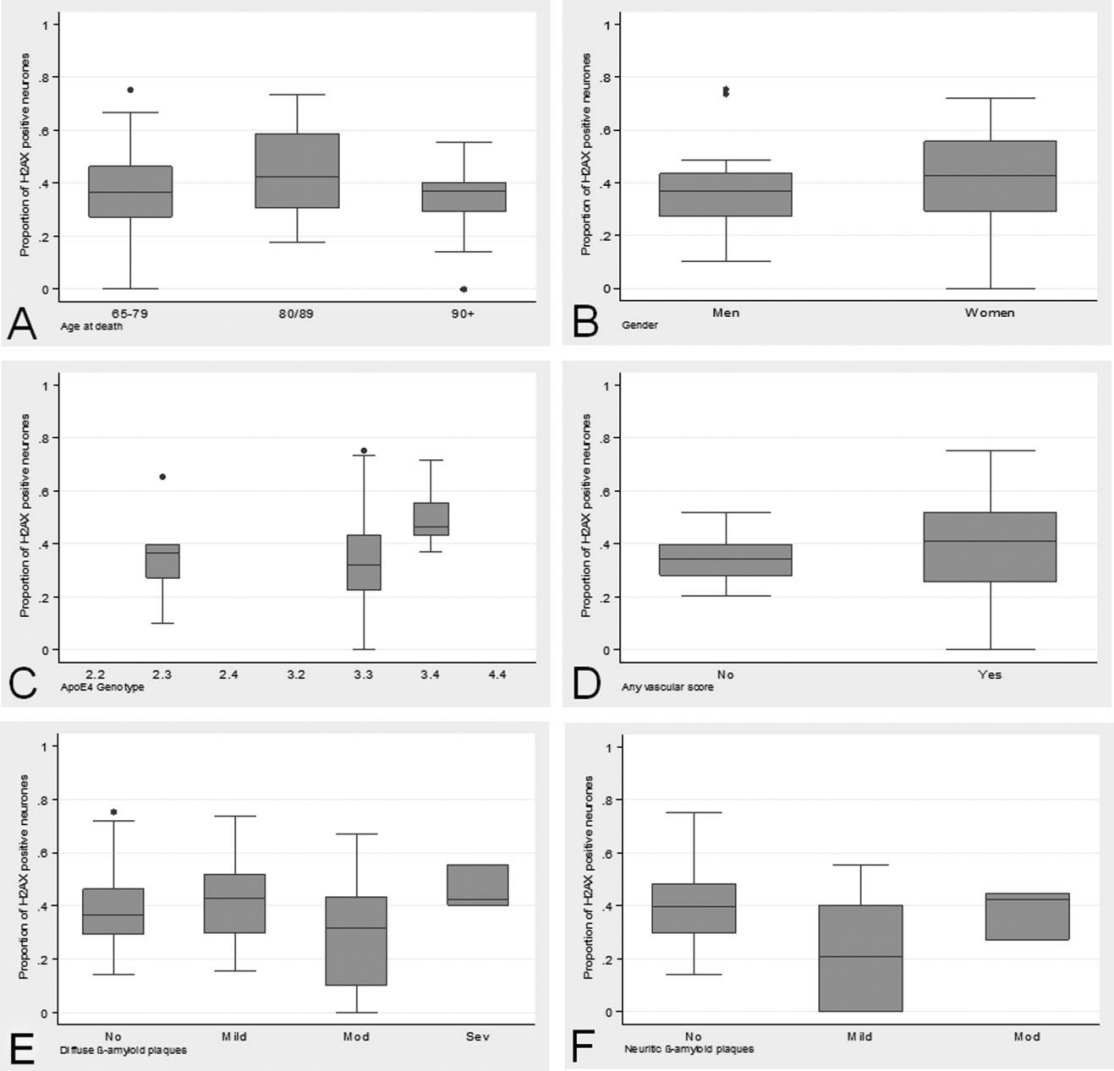
Association of neuronal DDR with clinical information. γH2AX ^+^ neurones did not show any association with (**A**) age, (**B**) sex, (**C**) ApoE genotype, (**D**) vascular pathology, (**E**) diffuse β‐amyloid plaques or (**F**) neuritic β‐amyloid plaques.

Neither γH2AX^+^ nor DNA‐PKcs^+^ neuronal counts (or proportion of immunopositive neurones) were related to age (Figure 4 **A**), sex (Figure 4 **B**), ApoE genotype (Figure 4 **C**), vascular pathology (Figure 4 **D**), diffuse β‐amyloid plaques (Figure 4 **E**) or neuritic β‐amyloid plaques (Figure 4 **F**) (all *P* values > 0.2). Furthermore, there was no association of either diffuse or neuritic β‐amyloid plaques to the relationship of DNA‐PKcs or γH2AX and the individual's last MMSE score (all *P* values > 0.1).

### Neuronal DNA damage is associated with the induction of senescence‐associated β‐galactosidase but not with activation of the classical senescence signalling pathway or apoptosis

**Figure 5:**
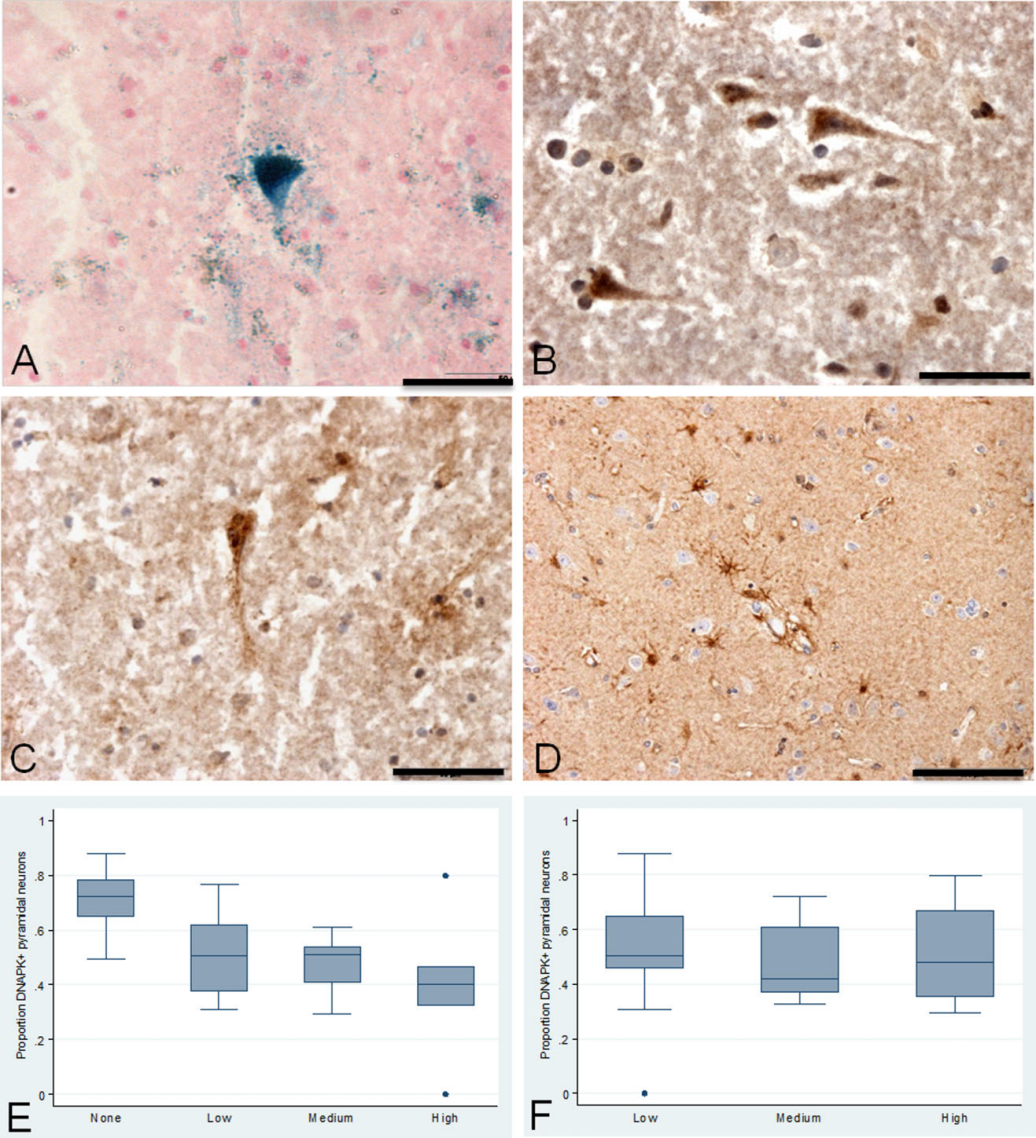
Expression of senescence and apoptosis‐associated markers. (**A**) SA‐β‐galactosidase^+^, (**B**) p53^+^ and (**C**) activated caspase‐3^+^ pyramidal neurones were detected in the ageing brain. (**D**) p16 was exclusively associated with astrocytes. Levels of (**E**) p53^+^ neurones negatively associated with DNA‐PKcs ^+^ neurones but not (**F**) caspase‐3^+^ neurones. Scale bar represents 50 μm in (**A–C**) and 100 μm in (**D**).

SA‐β‐gal^+^ pyramidal neurones and small cells were present throughout the FCx of the ageing brain (Figure 5 **A**). Inter‐rater scores quantitating SA‐β‐gal^+^ neurones agreed for 58% of cases, and for the remainder differed by one category (κ = 0.42, *P* < 0.001). Increasing levels of SA‐β‐gal^+^ pyramidal neurones were weakly associated with the total number of DNA‐PKcs^+^ neurones (*P* = 0.08), but there was no association with either the proportion of γH2AX^+^ neurones (counts and proportions *P* > 0.2) or DNA‐PKcs^+^ neurones (*P* = 0.13). p16 immunoreactivity was exclusively associated with astrocytes throughout the FCx (Figure 5 **B**). Relative scale measurements of p53^+^ pyramidal neurones showed a statistically significant declining trend with increasing DNA‐PKcs^+^ neurones (counts *P* = 0.007, proportions *P* = 0.006, Figure 5 **C,E**) but not γH2AX^+^ neurones (counts and proportions *P* > 0.2). No association between activated caspase‐3^+^ neurones and DNA‐PKcs^+^ or γH2AX^+^ neurones was detected (Figure 5 **D,F**), though numbers in the high group limit statistical power despite some increase with γH2AX^+^ neurones (all *P* values > 0.3).

## Discussion

Previous work in MRC CFAS and other population‐based studies have shown that individuals whose cognitive status is apparently discrepant when compared with conventional pathological correlates of dementia (plaques, tangles, vascular lesions, synucleinopathy) may represent as much as 20% of the aged population [1]. Both high pathology/normal cognition and low pathology/low cognition states are encountered [1]. The majority of dementia research focuses on cases selected on the basis of clinical information and single pathologies or clinicopathological diagnostic entities. Population‐based dementia studies, however, show that neuropathologies commonly coexist and combine to produce cognitive impairment and that the presence of classical hallmarks of Alzheimer‐type pathology, namely β‐amyloid plaques and neurofibrillary tangles, overlap so that classical pathologies provide an incomplete pathological explanation for dementia in the ageing population [1]. Population‐based studies are therefore essential to identify novel biomarkers and to develop new therapeutic strategies, complementing the case‐control approach [5]. The present population‐based study of individuals with early Braak and Braak (entorhinal stage) cases, in which tangles are restricted to mesial temporal lobe structures, demonstrates that individuals with high levels of a neuronal DDR are more likely to have lower MMSE scores. These findings suggest, in some individuals, that accumulation of DNA damage contributes to neuronal dysfunction and cognitive impairment independent of established Alzheimer pathology in the ageing brain. Furthermore, this study shows an association between the neuronal DDR and the expression of SA‐β‐gal in post‐mitotic neurones, but which is not associated with traditional senescence‐associated signalling molecules.

An imbalance between levels of pro‐oxidants and anti‐oxidants, in favour of the former, can result in oxidative stress [12]. The brain has a limited DNA repair capacity and is particularly susceptible to oxidative stress [28]. The oxidative stress theory of ageing proposes that a progressive and irreversible accumulation of reactive oxygen species (ROS) above the clearance capacity of the cell leads to DNA damage, mitochondrial dysfunction and eventually cell death [29, 30, 31, 32]. Oxidative stress can result in lipid peroxidation and the generation of relatively stable end‐products including MDA, and the oxidative modification of RNA and DNA bases. Several studies have demonstrated significantly increased levels of MDA in AD and MCI brains, particularly in regions associated with a high prevalence of Alzheimer‐type pathology [33, 34, 35], and a disease‐dependent increase in oxidative markers localized to synapses in post mortem FCx from MCI and late‐stage AD patients [36]. In contrast, other studies have shown that levels of lipid peroxidation markers, oxidized nucleosides and nucleotides inversely correlate with neurofibrillary tangle content [17, 18, 22, 37], suggesting that oxidative stress and oxidative damage to DNA and RNA is an early event in AD pathology. Our data demonstrate the extent of population variation in lipid peroxidation and the DDR at low Braak and Braak stages, and support the alternative model of neuronal loss in AD proposed by Nunomura *et al*., in which oxidative stress occurs before the formation of plaques and tangles and causes neuronal dysfunction [38], in the ageing population. It is likely that widespread DNA damage resulting from increased oxidative stress and a decreased ability to repair DNA damage triggers this neuronal dysfunction ultimately leading to cognitive impairment.

A significant correlation between levels of oxidative stress markers and MMSE has been previously reported [36], supporting this study's findings that individuals with high levels of a neuronal DDR are likely to have significantly lower MMSE scores and have dementia. The lack of significant association with dementia in the study likely reflects cohort size and a reduced power to detect an effect based on the comparison of 13 demented cases to 22 non‐demented cases. In contrast, an MMSE score was available for all the cohort. Although recent studies have demonstrated an increased vulnerability to oxidative damage in AD and MCI patients with the ApoEε4 allele [39, 40], we did not detect any correlation between the neuronal DDR and ApoE genotype in the cohort.

The detection of SA‐β‐gal^+^ neurones and their weak association with the total number of DNA‐PKcs^+^ neurones in the ageing brain supports a recent study that demonstrated that the accumulation of DNA damage can induce a senescence phenotype in mature, post‐mitotic neurones in ageing mice and confirm that senescence is not restricted to proliferating cells [23]. Senescent cells produce and secrete a range of molecules including pro‐inflammatory cytokines and pro‐oxidants [41] and can induce a DDR in neighbouring cells *in vitro* [42], suggesting a mechanism whereby this change in phenotype may underlie neuronal dysfunction in the ageing brain. This may operate either through dysfunction of the neurones themselves, where cells that appear viable may be ‘zombie cells’ that do not function normally, or by impairing their interaction with other cell types, such as the neuronal regulation of microglia.

Although DNA‐PKcs is constitutively expressed by most cell types [43], an increase in DNA‐PKcs expression is associated with neurones within infarcts [44], whereas a reduction in expression is associated with acute CNS injury due to ischaemia [45] and established AD, as recently reviewed [46]. Levels of DNA‐PKcs are regulated by post‐transcriptional and post‐translational mechanisms [43], including regulation by p53‐inducible gene 3 (PIG3) [47]. p16 and p53 play a critical role in the induction of cell senescence *in vitro*, as recently reviewed [10]. Overexpression of p16 induces cellular senescence [48, 49], whereas activation of the p53 signalling pathway may protect neurones from DNA damage through cell cycle arrest and the induction of DNA repair, or trigger cellular senescence or result in apoptosis [50]. However, in this study, we did not detect a relationship between the neuronal DDR and these traditional senescence‐associated signalling molecules. p16 was not expressed by neurones and was exclusively associated with cells with an astrocytic morphology. Neuronal p53 expression was inversely related to DDR, suggesting a response to persistent DNA damage in neurones involving decreased p53 levels to prevent apoptosis and promote cell survival. The signalling pathway(s) responsible for the induction of neuronal senescence and the role of neuronal senescence in the ageing brain remain unknown, but it is likely that this change in phenotype contributes to neuronal dysfunction, ultimately resulting in cognitive decline in the ageing population.

In research performed on post mortem material, the possibility of artefacts due to post mortem delay and/or brain pH must be considered. A study examining the effects of PMD on measures of oxidative damage reported similar levels between cases with a short post mortem interval and those with a longer interval [51]. We report that neither levels of oxidative stress nor the DDR were associated with PMD. However, DNA‐PKcs detection was associated with brain pH, but it should be noted that this was a positive association, the opposite of what would have been predicted if a poor premorbid state, which is accompanied by low brain pH, induced DNA‐PKcs expression.

In summary, we have defined the molecular signature of a neuronal DDR that significantly associates with cognitive impairment in older individuals with only early stage Alzheimer‐type pathology. We also demonstrate that this is associated with markers of a senescent phenotype in these post‐mitotic cells. As these individuals were at only the lowest Braak and Braak stages, this neuronal DDR is not attributable to established AD. It may be independent of Alzheimer's or interact with the earliest molecular stages of the disease. This remains to be resolved, but its association with cognition suggests that oxidative stress and the associated DDR is a potentially important line of investigation in its own right, rather that solely part of the pathogenesis of AD. Furthermore, cognitive decline in brain ageing may involve a complex interaction of specific pathological entities with ageing processes. Future work to characterize the effects of oxidative DNA damage on the neuronal molecular profile, and any interaction with similar processes in the glial cell populations, will potentially reveal novel dementia biomarkers, cellular pathways and specific therapeutic targets.

## Disclosure statement for authors

There are no conflicts of interest.

## Supporting information


**Figure S1.** Association of neuronal DDR with clinical information. DNA‐PKcs^+^ neurones did not show any association with (**A**) age, (**B**) sex, (**C**) ApoE genotype, (**D**) vascular pathology, (**E**) diffuse β‐amyloid plaques or (**F**) neuritic β‐amyloid plaques.
