# Supplementary figures and images for: A neuronal DNA damage response is detected at the earliest stages of Alzheimer's neuropathology and correlates with cognitive impairment in the Medical Research Council's Cognitive Function and Ageing Study ageing brain cohort
Source: Neuropathol Appl Neurobiol. 2015 Apr 23;41(4):483–96. doi: 10.1111/nan.12202 (PMC4861215; doi:10.1111/nan.12202)

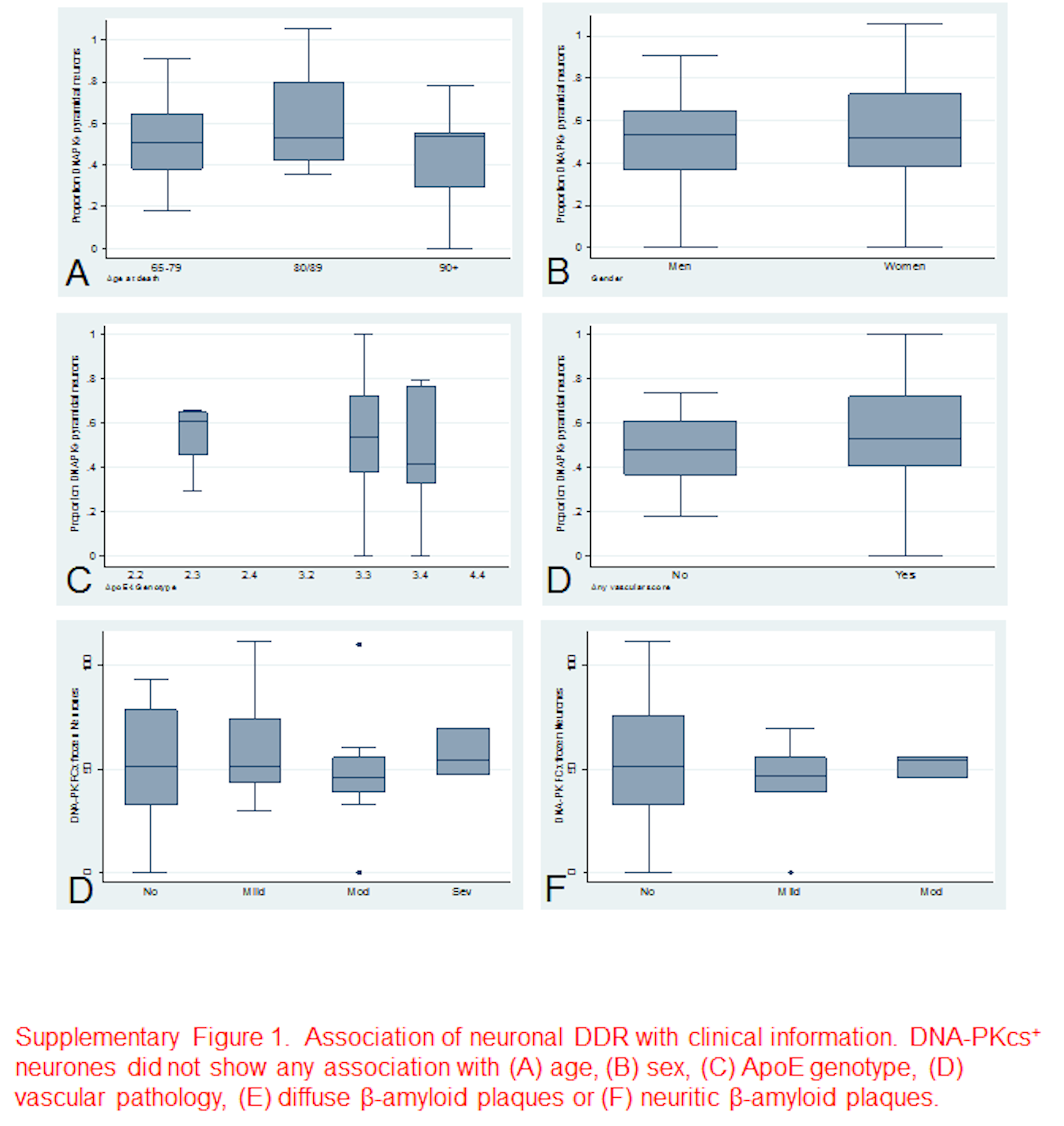

Supplement: Supplementary file 1 — Figure S1. Association of neuronal DDR with clinical information. DNA‐PKcs+ neurones did not show any association with (A) age, (B) sex, (C) ApoE genotype, (D) vascular pathology, (E) diffuse β‐amyloid plaques or (F) neuritic β‐amyloid plaques. [file NAN-41-483-s001.tif]
